# The central vein sign and paramagnetic rim lesions: biomarkers for an accurate differential diagnosis between multiple sclerosis and migraine

**DOI:** 10.1590/0100-3984.2025.0015

**Published:** 2025-09-22

**Authors:** Viviana Regina Konzen, Alessandro Finkelsztejn, Raquel Prates dos Santos, Adolfo Moraes de Souza, Matheus de Lima Ruffini, Renata Gomes Londero, Juliana Ávila Duarte

**Affiliations:** 1 Universidade Federal do Rio Grande do Sul (UFRGS), Porto Alegre, RS, Brazil; 2 Hospital de Clínicas de Porto Alegre (HCPA), Porto Alegre, RS, Brazil

**Keywords:** Cerebral veins/diagnostic imaging, Multiple sclerosis/diagnosis, Migraine disorders/diagnosis, Neuroimaging/methods, Magnetic resonance imaging/methods, Biomarkers/analysis., Veias cerebrais/diagnóstico por imagem, Esclerose múltipla/diagnóstico, Transtornos de enxaqueca/diagnóstico, Neuroimagem/métodos, Ressonância magnética/métodos, Biomarcadores/análise.

## Abstract

**Objective:**

This study aimed to assess whether the evaluation of the central vein sign (CVS) and paramagnetic rim lesions (PRLs) using susceptibility-weighted magnetic resonance imaging (MRI) can distinguish multiple sclerosis (MS) from migraine.

**Materials and Methods:**

In this single-center observational study, we conducted a cross-sectional analysis of the CVS, determining the proportion of CVS-positive lesions per individual and absolute counts, using thresholds of 3 lesions (select3*) and 6 lesions (select6*), and of PRLs in participants with MS and in those with migraine, from 3.0-T MRI brain scans.

**Results:**

The study included 20 participants with MS, 20 with migraine, and 20 included as healthy controls. The proportion of participants with CVS-positive lesions was higher in the MS group than in the migraine group (61.8% vs. 10.4%), and PRLs were observed exclusively in the MS group. The presence of at least one PRL and the select6* criterion demonstrated the highest diagnostic accuracy within the study sample.

**Conclusion:**

The detection of the CVS and of a PRL on 3.0-T MRI scans may serve as a reliable biomarker to differentiate MS from migraine.

## Abstract (pt)

**Objetivo:**

Este estudo teve como objetivo avaliar se a análise do sinal da veia central (SVC) e das lesões com anel paramagnético (LAP) em imagens de ressonância magnética (RM) baseadas em suscetibilidade pode diferenciar esclerose múltipla (EM) de migrânea.

**Materiais e Métodos:**

Em um estudo observacional de centro único, realizamos uma análise transversal do SVC avaliando a proporção de lesões com SVC por indivíduo e a contagem absoluta de lesões utilizando os critérios de 3 (select3*) e 6 (select6*) lesões e LAP em participantes com EM e migrânea, utilizando exames cerebrais de RM de 3-T.

**Resultados:**

O estudo incluiu 20 participantes com EM, 20 com migrânea e 20 controles saudáveis. A proporção de lesões apresentando SVC foi maior em indivíduos com EM (61,8%) do que em pacientes com migrânea (10,4%). As LAPs foram identificadas exclusivamente em pacientes com EM. A presença de pelo menos uma LAP e o critério select6* apresentaram o melhor desempenho diagnóstico nessa amostra.

**Conclusão:**

A identificação do SVC e das LAPs em exames de RM de 3-T pode ser um biomarcador confiável para diferenciar EM de migrânea.

## INTRODUCTION

Multiple sclerosis (MS) is the most common nontraumatic disabling disease affecting young adults^**(^1^)**^. In recent decades, new diagnostic criteria have been developed to facilitate early diagnosis of the disease^**(^2^)**^. However, misdiagnosis is a persistent problem, exposing patients to unnecessary medical risks and morbidity^**(^3^)**^.

Magnetic resonance imaging (MRI) plays a central role in the diagnosis of MS, and new radiological biomarkers such as the central vein sign (CVS) and paramagnetic rim lesions (PRLs) have recently gained attention for improving diagnostic specificity^**(^4^)**^. The CVS reflects the perivenular development of inflammatory demyelination, whereas a PRL indicates chronic perilesional inflammation characterized by iron-laden microglia/macrophages at the lesion edge^**(^5^,^6^)**^. Several criteria have been proposed for evaluating CVS positivity, including thresholds of 35-50% of lesions^**(^7^)**^ and simplified approaches such as the use of the select6* and select3* algorithms^**(^8^,^9^)**^.

For the diagnosis of MS, PRLs have high specificity^**(^10^)**^. In rare cases, PRLs can be observed in other conditions associated with chronic neuroinflammation, such as neuromyelitis optica spectrum disorder and Susac syndrome. However, one major limitation of PRLs is their low sensitivity; many patients with MS do not exhibit PRLs on routine MRI scans^**(^11^)**^.

Among the differential diagnoses of MS-like symptoms, migraine deserves particular attention. It is a prevalent condition that may present with white-matter lesions (WMLs) on MRI, with clinical and radiological features that sometimes mimic those of MS^**(^12^,^13^)**^. In addition, migraine frequently coexists with MS, with reported prevalence rates of up to 31% among patients with MS^**(^14^)**^.

Although migraine and MS can both manifest as WMLs, differences exist in the distribution and characteristics of the lesions. Migraine-related lesions are usually small and few in number, with a predominantly subcortical distribution, whereas MS-related lesions tend to be larger and more numerous, being distributed throughout the periventricular, juxtacortical, and infratentorial regions. In addition, migraine-related lesions rarely exhibit markers such as the CVS and PRLs, which are more specific to MS. Incorporating the evaluation of these biomarkers into imaging assessment could enhance diagnostic accuracy.

To distinguish between MS-related and migraine-related lesions on MRI, it is essential to evaluate populations with each disease separately, aiming to identify the most reliable biomarkers and to understand the specific contributions of each disease to the MRI findings. Patients with cardiovascular risk factors that could lead to MRI abnormalities should also be evaluated separately.

The aim of the present study was to evaluate MRI lesions and determine the diagnostic value of radiological biomarkers in patients with MS and in those with migraine.

## MATERIALS AND METHODS

This was a cross-sectional study that included participants recruited from the Neuroimmunology Outpatient Clinic and Headache Outpatient Clinic of the Hospital de Clínicas de Porto Alegre (HCPA), a tertiary care university hospital in the city of Porto Alegre, Brazil. The study was approved by the Research Ethics Committee of the HCPA (Reference no. GPPG 2019-0287), and all participants gave written informed consent.

### Participant selection

Patients with MS, diagnosed on the basis of the 2017 revision of the McDonald diagnostic criteria^**(^15^)**^, without migraine, as determined by applying the criteria established in the International Classification of Headache Disorders^**(^16^)**^, were included. Patients with MS who had previously undergone a 3.0-T MRI brain scan were recruited consecutively from the Neuroimmunology Outpatient Clinic of the HCPA. Disability status was determined from the Expanded Disability Status Scale (EDSS) score recorded in the medical record at the time closest to MRI acquisition.

The second group comprised patients diagnosed with migraine, with and without aura, according to the definition established in the International Classification of Headache Disorders^**(^16^)**^, including only patients in whom a previous 3.0-T MRI brain scan had demonstrated at least one WML. Patients with migraine were recruited consecutively from the Headache Outpatient Clinic of the HCPA. Pregnant individuals were excluded from the MS and migraine groups, as were those with a history of hypertension, diabetes, traumatic brain injury, stroke, or neurosurgical intervention, as well as those who were current smokers. The control group was composed of healthy individuals who had previously undergone a 3.0-T MRI brain scan at the HCPA, and the images were collected from a normal imaging database managed by the Neuroradiology Department of the hospital.

### MRI scan acquisition

Patients in the MS group underwent brain MRI, with fluid-attenuated inversion recovery (FLAIR) susceptibility-weighted imaging (SWI) sequences and gadolinium contrast injection, in accordance with the routine neuroimmunology clinic protocol, between December 2019 and February 2023, and the resulting images were analyzed retrospectively. Patients in the migraine group underwent prospective scanning, in accordance with the same protocol, between September and November of 2021. Participants in the control group were scanned in accordance with the same protocol between February 2020 and January 2023, and the resulting images were analyzed retrospectively.

All MRI examinations were performed in a 3.0-T scanner (Ingenia; Philips Medical Systems, Best, the Netherlands). A standardized imaging protocol, including conventional and SWI sequences, was used. Conventional imaging without contrast enhancement included axial T2*-weighted imaging (T2*WI)-repetition time/echo time (TR/TE) = 3,000/80 ms; matrix = 576 × 576; field of view (FOV) = 230 × 185 mm; slice thickness/interslice gap = 4.0/1.0 mm; coronal T2*WI-TR/TE = 3,000/90 ms; matrix = 200 × 172; FOV = 110 × 110 mm; slice thickness/interslice gap = 2.0/0.2 mm; and axial three-dimensional FLAIR-TR/TE = 4,800/302 ms; inversion time (TI) = 1,650 ms; matrix = 256 × 256; FOV = 256 × 256 × 180 mm; voxel size = 1.0 × 1.0 × 1.0 mm^3^; slice thickness/interslice gap = 1.0/0 mm; acceleration factor (sensitivity encoding) = 2.5 × 2.5. The SWI protocol included the acquisition of magnitude and phase images. The imaging parameters for SWI were as follows: TR/TE = 27/20 ms, voxel size = 0.9 × 0.9 × 1.5 mm^3^, slice thickness = 1.5 mm (no interslice gap), and FOV = 230 × 185 mm. When clinically indicated, 0.1 mmol/kg of the gadolinium-based contrast agent gadopentetate dimeglumine (Omniscan; GE Healthcare, Milwaukee, WI, USA) was administered in accordance with institutional protocols.

### MRI analysis

Two trained neuroradiologists from the HCPA, working independently, evaluated the images. One had over 10 years of experience as a staff neuroradiologist, and the other had over 20 years of academic expertise with a special focus on neuroimmunology.

The number of lesions, the presence of a CVS, and the presence of PRLs were assessed on SWI sequences. Specifically, PRLs were evaluated on SWI phase images, which were available for all patients, for all discrete WMLs ≥ 3 mm in diameter. The WMLs were categorized according to their topographical location in the brain: periventricular white matter; subcortical white matter; deep white matter (DWM); cortical region; or infratentorial region.

In all of the scans, two trained raters, working independently, evaluated the location and characteristics of WMLs, as well as determining positivity for the CVS and PRL biomarkers. Both raters were blinded to the identity of the participant and to the analysis of the other rater.

**Figure 1:**
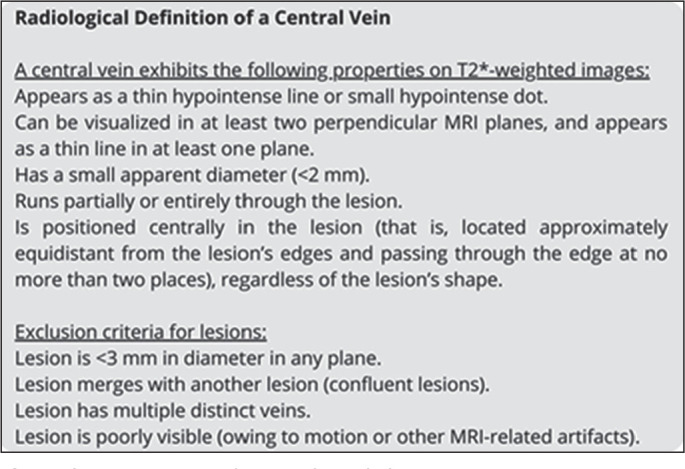
Radiological definition of the CVS according to the criteria established by the North American Imaging in Multiple Sclerosis Cooperative^(^17^)^.

The analysis of the CVS was based on the criteria established by the North American Imaging in Multiple Sclerosis Cooperative^**(^17^)**^, as detailed in Figure 1. The proportion of brain lesions that were perivenular was calculated for each participant, referred to as the “proportion of CVS-positive lesions”. The positivity for CVS was also assessed with the select3* and select6* algorithms.

For the select6* algorithm, a scan was classified as CVS-positive if it revealed at least six morphologically characteristic lesions with central veins or if there were fewer than six such lesions but the number of CVS-positive lesions exceeded the number of CVS-negative lesions. If neither condition was met, the scan was considered select6*-negative. For the select3* algorithm, a scan was classified as CVS-positive if it revealed at least three candidate lesions meeting the CVS criteria. Scans with fewer than three candidate lesions were classified as select3*-negative.

A chronic lesion was classified as a PRL if it met the following criteria: having a partially or completely hypointense rim relative to the lesion core and surrounding white matter; alignment of the rim with the lesion edge on FLAIR imaging; and visibility of the rim on at least two consecutive slices. The PRLs that did not correspond to lesion edges or hypointense areas on FLAIR were excluded. In addition, precautions were taken to avoid misidentifying as PRLs veins or signals from the white/gray matter border.

### Sample size

A sample size of 32 subjects (16 per group) was calculated to test whether there is a difference minimum of 8.2973 measurement units (one standard deviation) in the means of Y between the study and control groups (with an increase of 10% for possible losses and refusals, this number should be 36). The calculation considered a power of 80%, a significance level of 5%, and a standard deviation of 8.2973 measurement units^**(^18^)**^. The calculation was carried out by using the PSS Health online tool (R Foundation for Statistical Computing, Vienna, Austria).

### Statistical analysis

The data were entered into Excel and subsequently imported into RStudio for statistical analysis using the R programming language (R Foundation for Statistical Computing). The normality of the variables was assessed with the Shapiro-Wilk test. The variables presented an asymmetrical distribution and are therefore expressed as median and range, being compared between groups by using the Kruskal-Wallis test. For multiple comparisons, the Dunn-Bonferroni test was used. The variables with normal distribution were compared by using the analysis of variance test. The categorical variables are expressed as absolute frequencies and percentages, with associations being quantified with the chi-square test followed by the z-test for proportions. A significance level of 5% was adopted. Diagnostic performance metrics, including sensitivity, specificity, positive predictive value, negative predictive value, and accuracy, were calculated by using standard formulas. These measures were determined by comparing patients with MS group with the combined group of patients with migraine and healthy controls, the comparison being based on the presence or absence of the CVS and PRLs.

## RESULTS

The study sample included 20 patients in the MS group, 20 patients in the migraine group, and 20 healthy subjects in the control group. Among the patients in the MS group, 10 (50%) were women, the mean age was 39.3 ± 14.5 years, and the median disease duration was 5 years (range, 0-20 years). The MS group was characterized by predominantly moderate clinical disability, as evidenced by the EDSS score (mean, 3.75 ± 2.07). Among the patients with migraine, 15 (75%) were women and the mean age was 42.5 ± 11.8 years. Table 1 presents the clinical and sociodemographic characteristics of the MS, migraine, and control groups. Although no formal matching was performed, the groups were comparable in age, with no statistically significant difference among them (*p* = 0.459). In contrast, there was a significant difference in sex distribution, with a predominance of women in the migraine group (*p* = 0.001).

**Table 1:** Clinical and sociodemographic characteristics of the participants, by group.

Characteristic	Group			p^*^
	MS (n = 20)	Migraine (n = 20)	Control (n = 20)	
Age (years), mean ± SD	39.3 ± 14.5	42.5 ± 11.8	37.0 ± 11.5	0.459^*^
Sex, n (%)				
Female	10 (50)^a^	15 (75)^b^	8 (40)^a^	0.00†
Male	10 (50)	5(25)	12 (60)	
MS subtype, n (%)				
Relapsing-remitting	18 (90)	NA	NA	
Primary progressive	1(5)	NA	NA	
Secondary progressive	1(5)	NA	NA	
EDSS score, mean ± SD	3.75 ± 2.07	NA	NA	
Disease duration (years),				
median (range)	5 (0-20)	NA	NA	

* Analysis of variance.

† Chi-square test followed by the z-test for proportions.

a,b Different letters represent statistically significant differences.

NA, not applicable.

In the MS group, all of the patients had WMLs, of which a total of 1,697 were identified. In the migraine group, 14 patients (70%) patients had WMLs and a total of 96 WMLs were identified. None of the healthy controls had WMLs. Table 2 presents the number and characteristics of WMLs in each of the three groups.

**Table 2:** Numbers and characteristics of the lesions among the participants, by group.

Variable	Group		
	MS (n = 20)	Migraine (n = 20)	Control (n = 20)
Presence of lesions, n (%)	20 (100)	14 (70)	0(0)
Total number of lesions	1,697	96	0
Lesions per participant, median (range)	69 (22-221)	5(0-23)	0
CVS-positive lesions			
Total, n (%)	1,048 (61.8)	10 (10.4)	0
Range (%-%)	20.9-94.9	0-100^*^	0
Number per participant, median (range)	37.5(12-135)	0(0-3)	0
PRLs			
Participants with ≥ 1 PRL, n (%)	20 (100)	0	0
Total, n (%)	605 (35.7)	0	0
Range (%-%)	6.3-82.8	0	0
Number per participant, median (range)	13.5 (2-130)	0	0

* One patient had only two lesions, both of which showed the CVS.

**Figure 2:**
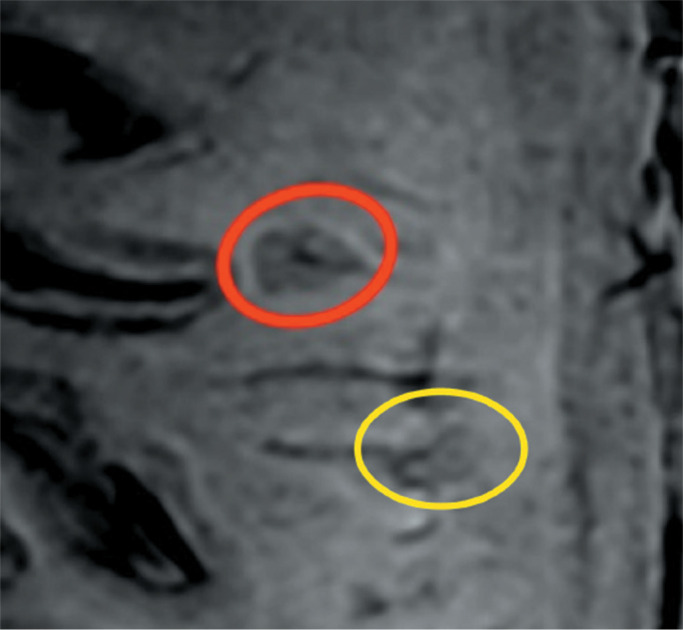
SWI magnitude image for illustrative purposes. Red circle: hypointense demyelinating lesion with the CVS. Yellow circle: hypointense rim corresponding to a PRL. Although this figure shows a magnitude image, PRLs in our study sample were evaluated on SWI phase images.

**Figure 3:**
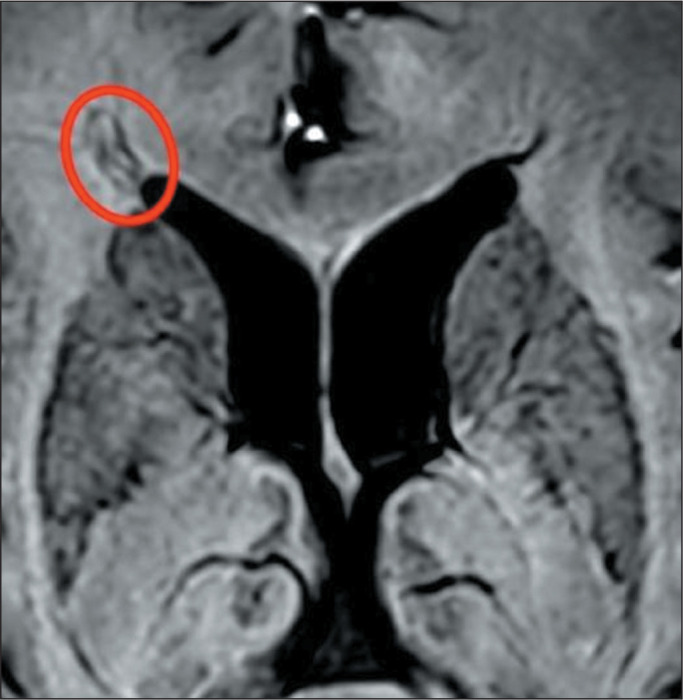
Axial T2-weighted SWI magnitude image: hypointense iron deposit ring (PRL) around an demyelinated lesion adjacent to the right anterior horn of the lateral ventricle.

**Figure 4:**
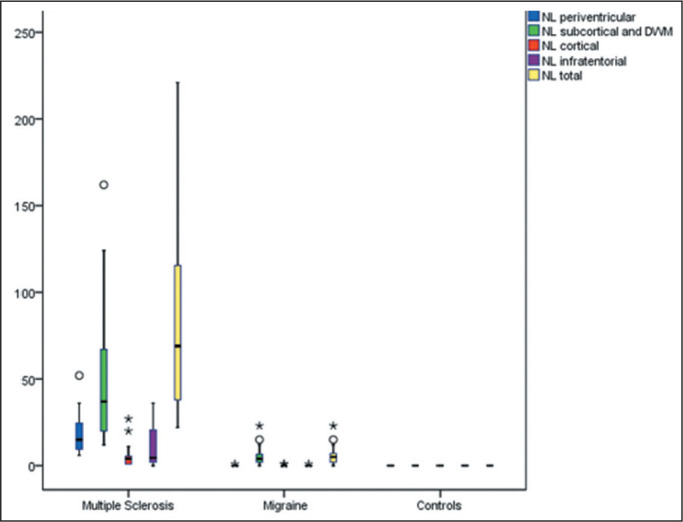
Box plot of the number of lesions (NL) among the groups evaluated, by lesion location.

**Figure 5:**
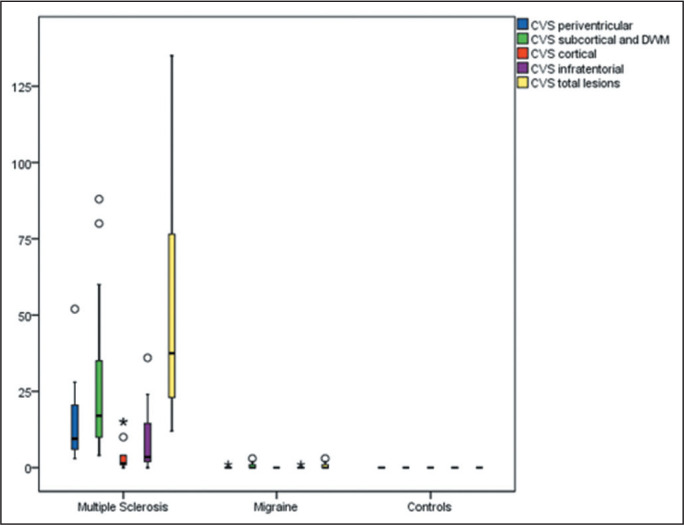
Box plot of the number of CVS-positive lesions among the groups evaluated, by lesion location.

Representative lesions, including one with a CVS, are illustrated in Figures 2 and 3. As shown in Table 2, Figure 4, and Figure 5, the proportion of CVS-positive lesions was higher in the MS group than in the migraine group (61.8% vs. 10.4%).

* *P* < 0.05. ** *P* < 0.01.

* *P* < 0.05.

**Figure 6:**
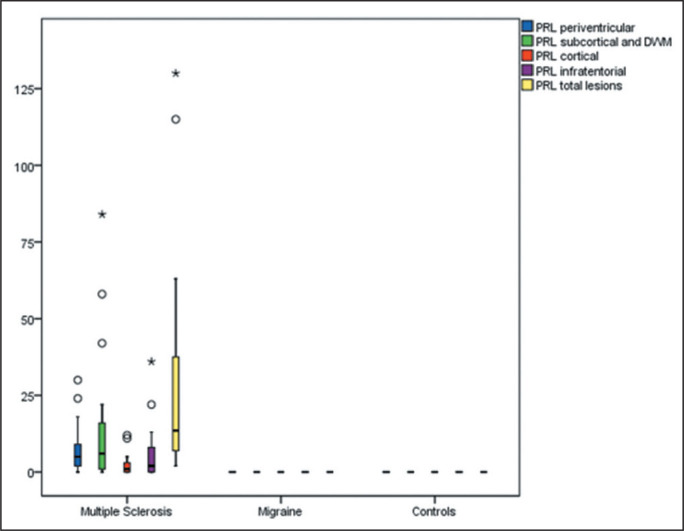
Box plot of the number of PRLs among the groups evaluated, by lesion location.

All of the patients in the MS group had at least one PRL, whereas no PRLs were identified in the migraine and control groups. Of the 1,697 lesions identified in the MS group, 605 (35.7%) were PRLs, with a median per patient of 13.5 PRLs. Figures 2 and 3 show distinctive examples of PRLs, and Figure 6 shows the distribution of PRL locations across the groups.

* *P* < 0.05.

When comparing the total number of lesions, CVS positivity, and PRL presence, we found statistically significant differences in terms of the brain regions affected. The total numbers of lesions, CVS-positive lesions, and PRLs-in the periventricular, subcortical, cortical and infratentorial regions-were greatest in the MS group. The numbers of lesions identified in the migraine group were statistically equal to those identified in the control group, with the exception of the numbers of subcortical and DWM lesions, which were higher in the migraine group. We found CVS-positive lesions in all of the MS group patients. In the migraine group, such lesions were rare and, when present, were located primarily in the subcortical region or DWM. In our study sample, PRLs were identified only in the MS group. Table 3 details these comparisons.

**Table 3:** Locations of the lesions evaluated, by brain region and group.

Group			Control	p^*^
Brain region	MS	Migraine		
All lesions^†^, median (range)				
Periventricular	15 (6-52)^a^	O(O-l)^b^	0 (O-O)^b^	< 0.001
Subcortical/DWM	37 (12-162)^a^	4 (0-23)^b^	0 (0-0)^c^	< 0.001
Cortical	4(l-27)^a^	O(O-l)^b^	0 (O-O)^b^	< 0.001
Infratentorial	4.5 (0-36)^a^	O(O-l)^b^	0 (O-O)^b^	< 0.001
Total	69 (22-221)^a^	5 (0-23)^b^	0 (0-0)^c^	< 0.001
CVS-positive lesions^†^, median (range)				
Periventricular	9.5 (3-52)^a^	O(O-l)^b^	0 (O-O)^b^	< 0.001
Subcortical/DWM	17 (4-88)^a^	0 (0-3)^b^	0 (O-O)^b^	< 0.001
Cortical	1.5 (0-15)^a^	0 (0-0)^b^	0 (O-O)^b^	< 0.001
Infratentorial	3.5 (0-36)^a^	O(O-l)^b^	0 (O-O)^b^	< 0.001
Total	37.5(12-135)^a^	0 (0-3)^b^	0(0-0^b^	< 0.001
PRLs^^1^^, median (range)				
Periventricular	5 (0-30)^a^	0 (0-0)^b^	0 (O-O)^b^	< 0.001
Subcortical/DWM	6 (0-84)^a^	O(O-O)^b^	0 (O-O)^b^	< 0.001
Cortical	l(0-12)^a^	0(0-0)^b^	0 (O-O)^b^	< 0.001
Infratentorial	2 (0-36)^a^	0 (O-O)^b^	0 (O-O)^b^	< 0.001
Total	13.5 (2-130)^a^	0 (0-0)^b^	0 (O-O)^b^	< 0.001

* Kruskal-Wallis test followed by the Dunn-Bonferroni test for multiple comparisons.

† Per patient.

a,b,c Different letters represent statistically significant differences.

Among the biomarker criteria, the presence of at least one PRL and the select6* CVS criterion showed the best diagnostic performance, followed by the select3* CVS criterion. The highest sensitivity was achieved when there was at least one PRL, three or more CVS-positive lesions, or six or more CVS-positive lesions. The highest specificity was achieved when there was at least one PRL or six or more CVS-positive lesions. Table 4 shows the performance of different CVS and PRL criteria in the diagnosis of MS.

**Table 4:** Diagnostic performance of the CVS and PRLs as biomarkers for the diagnosis of MS, by criterion.

Criterion	Sensitivity	Specificity	PPV	NPV	Accuracy
Select3^*^	100.0%	97.1%	95.2%	100.0%	98.2%
Select6^*^	100.0%	100.0%	100.0%	100.0%	100.0%
CVS threshold					
30%	95.0%	78.6%	86.4%	91.7%	88.2%
35%	90.0%	78.6%	85.7%	84.6%	82.4%
40%	80.0%	78.6%	84.2%	73.3%	79.4%
50%	70.0%	92.9%	93.3%	68.4%	79.4%
> 1 PRL	100.0%	100.0%	100.0%	100.0%	100.0%

PPV, positive predictive value; NPV, negative predictive value.

## DISCUSSION

In this cross-sectional, single-center study, we evaluated the presence of brain lesions, the CVS, and PRLs on 3.0-T MRI and investigated their diagnostic performance in differentiating between MS and migraine.

We found the incidence of the CVS on T2*WI at the level of individual lesion to be 61.8% in the participants with MS, comparable to the 40-92% reported in the literature^**(^19^)**^. Of the participants in our migraine group, 10.4% had a CVS-positive lesion, compared with 22% in a previous study of MS and migraine^**(^20^)**^. Other studies that specifically evaluated patients with migraine also reported a low prevalence of CVS-positive lesions among such patients. In a recent study, Cagol et al.^**(^21^)**^ found the median proportion of individuals with migraine who had CVS-positive lesions to be only 0.95% (interquartile range, 0.0-18.2%). In a broader sample of patients without MS, including those with migraine or other conditions, Sinnecker et al.^**(^7^)**^ reported a median proportion of CVS positivity per patient of 0% (range, 0-100%), reinforcing the idea that the CVS in non-MS disorders is generally rare. As expected, the median proportion of individuals with CVS-positive lesions was higher in our MS group than in our migraine and control groups (37.5% vs. 0% and 0%, respectively).

We found PRLs to be highly specific to the MS population, given that no PRLs were identified in the other groups and that all of our patients with MS had at least one PRL, as previously described^**(^10^,^11^)**^. We find it interesting that the prevalence of PRLs was higher in our MS group, in which 35.7% of all lesions had a PRL, with a median per patient of 13.5. Although these values are higher than those reported elsewhere^**(^10^,^11^)**^, they are in accordance with those reported in a recent systematic review and meta-analysis^**(^22^)**^. This discrepancy might be explained by the fact that the participants in our MS group had greater disability (as assessed with the EDSS) and a relatively short disease duration, because some PRLs fade over time and because a PRL is a predictor of disability accrual^**(^5^,^23^,^24^)**^. That further corroborates the potential role of PRL in driving clinical progression at a young age or a relatively short disease duration.

In our MS and migraine groups, the total number of lesions, as well as the prevalence of the CVS and PRLs, were higher among the subcortical and DWM lesions. That finding is in agreement with those of previous studies^**(^11^,^21^)**^.

In terms of diagnostic performance, the 40% CVS threshold demonstrated lower accuracy in the present study than in previous studies. However, in our sample, the simplified CVS assessment criteria, including the select3* and select6* algorithms, which streamline the evaluation process, showed the potential for greater diagnostic accuracy compared with the more time-intensive analysis of all lesions^**(^9^)**^. The discrepancy between our results and others in the literature may be attributed to the small sample size. However, our findings underscore the potential value of the CVS and PRLs as reliable biomarkers for distinguishing MS from migraine on MRI.

Our study has some limitations, one of which is the small sample size. Our use of 3.0-T MRI to evaluate radiologic biomarkers could also be viewed as a limitation because, although it increased the ability to identify the CVS and PRLs, 3.0-T MRI scanners are not readily available at many clinical facilities. One small study showed that 1.5-T and 3.0-T MRI are comparable in terms of their ability to identify PRLs^**(^25^)**^. Some studies have shown that the proportion of detectable CVS-positive lesions is lower when 1.5-T MRI is used^**(^26^)**^. Therefore, it is important for future studies to evaluate the performance of the CVS and PRL biomarkers when 1.5-T scanners are used in order to differentiate MS from migraine. Another potential limitation of our study is that our migraine group was composed predominantly of women. That was because the source of patients with migraine were recruited from the migraine clinic of a tertiary care hospital, where the majority of patients were women and most of the eligible men declined to participate. Although strict exclusion criteria were applied to minimize potential confounders, our findings cannot yet be generalized beyond the current study population, highlighting the need for further evaluation in individuals with MS or migraine who have multiple comorbidities that may contribute to the development of WMLs. In addition, most of the participants in our study had relapsing-remitting MS, which limited our ability to analyze lesion characteristics in the primary progressive MS and secondary progressive MS subtypes. Furthermore, we did not investigate the potential impact of disease-modifying treatments on lesion appearance, including the CVS and PRLs. Moreover, the disease duration was not systematically recorded among the participants with migraine. Because the time since the onset of migraine symptoms may influence MRI findings, future studies should aim to include this information in order to better characterize its potential impact. Another major consideration is that our MS population was recruited from the specialized MS outpatient clinic of a tertiary care hospital within the Brazilian Unified Health Care System. That setting typically includes patients with more severe disease, limited access to high-efficacy MS treatments, and restricted availability of MRI, which may have contributed to the higher degree of disability in our patient sample.

In conclusion, the presence of at least one PRL, along with the simplified select3* and select6* algorithms-which are more practical for routine clinical use-demonstrated strong diagnostic performance, with high specificity and sensitivity for accurately distinguishing MS from migraine. These findings are in line with the recently proposed updates to the McDonald criteria for MS, presented at the 2024 conference of the European Committee for Treatment and Research in Multiple Sclerosis^**(^27^)**^, which emphasize the role that MRI biomarkers such as the CVS and PRLs play in improving diagnostic accuracy.
